# A New Time-varying Concept of Risk in a Changing Climate

**DOI:** 10.1038/srep35755

**Published:** 2016-10-20

**Authors:** Ali Sarhadi, María Concepción Ausín, Michael P. Wiper

**Affiliations:** 1 Department of Civil and Environmental Engineering, University of Waterloo, Waterloo, Ontario, N2L-3G1, Canada; 2 Departamento de Estadística, Universidad Carlos III de Madrid, Getafe, Spain

## Abstract

In a changing climate arising from anthropogenic global warming, the nature of extreme climatic events is changing over time. Existing analytical stationary-based risk methods, however, assume multi-dimensional extreme climate phenomena will not significantly vary over time. To strengthen the reliability of infrastructure designs and the management of water systems in the changing environment, multidimensional stationary risk studies should be replaced with a new adaptive perspective. The results of a comparison indicate that current multi-dimensional stationary risk frameworks are no longer applicable to projecting the changing behaviour of multi-dimensional extreme climate processes. Using static stationary-based multivariate risk methods may lead to undesirable consequences in designing water system infrastructures. The static stationary concept should be replaced with a flexible multi-dimensional time-varying risk framework. The present study introduces a new multi-dimensional time-varying risk concept to be incorporated in updating infrastructure design strategies under changing environments arising from human-induced climate change. The proposed generalized time-varying risk concept can be applied for all stochastic multi-dimensional systems that are under the influence of changing environments.

In infrastructure designs and water resources planning, the term risk is defined as the probability of failure of a water system over a planning horizon. The failure may occur under the impact of extreme rare hydro-climate events. To estimate the reliability of the system over its lifetime, the exceedance probability of rare events should be estimated. Current engineering planning and infrastructure designs are based on the fundamental stationary risk concepts, which assume that the occurrence probability of extreme events is not expected to change over time, and that past records can represent future climate events^1^,^2^. Under stationarity, it is also assumed that the nature of extreme climate events will not change over time. Many of these assumptions may be challenged by changes arising from human activities^3^,^4^. Anthropogenic effects are expected to intensify the natural variability, probability of occurrence, and frequency of extreme climate events over time. These changes are termed non-stationarity in the literature^5^,^6^,^7^. Anthropogenic greenhouse gas emissions are changing the atmospheric composition leading to global warming. Subsequent increased temperature enhances atmosphere water vapor—as the main greenhouse gas—which can increase the incidence of extreme hydro-climatic phenomena^8^. Global warming is directly increasing the risk of extreme climate events, such as tornados, rainstorms, floods, and droughts—all of which are becoming more severe and frequent^8^,^9^. These events have caused major human suffering and economic damages around the world in recent record-breaking warm years^10^. An increased number of extreme climate events raise the question of how well water resources infrastructure can deal with these nature-changing extreme disasters in the changing modern climate. In this environment, to strengthen the reliability of infrastructure designs and the management of water systems, water professionals must revise current risk-based planning strategies to reflect anomalies in extreme climate events that may result from the impact of human-induced climate change. Water associated communities therefore need to adopt a new adaptive time-varying, or non-stationary, risk framework for water resources planning and infrastructure designs. With respect to the increasing frequency of extreme climatic events and their changing nature over time, this study argues that the risk concept should be updated from a static stationary concept to a flexible time-varying one. Applying the new concept, one is able to adapt the design and management of water systems to the dynamic anthropocentrically-forced conditions. There are many studies in the context of one-dimensional time varying modeling in water systems risk assessment^11^,^12^,^13^. Natural climate-associated processes, however, are multi-dimensional phenomena, and are characterized with multi-attributes that are statistically dependent^14^. Accordingly, if analytical risk approaches are designed based on only one dimension of the climate processes, it may lead to high uncertainty for infrastructure designs, and destructive consequences in water resources systems. It is hence of crucial importance to adopt a multi-dimensional perspective in risk analyses to realistically include all the correlated dimensions simultaneously.

Several publications have appeared in recent years after introducing copulas in hydrology and geosciences to model multi-dimensional hydro-climate processes^15^,^16^,^17^,^18^, and concurrent climate extremes^19^,^20^. These studies, and similar ones, ignore time-varying non-stationarity in the multivariate risk analysis under changing environments. Due to the lack of a robust methodology, and the complexity of the theoretical concepts, the notion of multi-dimensional time-varying risk is new and scarcely investigated in the water resources area and other disciplines. The present study assesses the effect of dynamic climate change conditions on the risk of extreme multi-dimensional events over time for an illustrative climate example. It also compares the results with the currently used multi-dimensional static stationary techniques. For doing so, a generalized fully flexible multi-dimensional time-varying risk framework is proposed that evolves through time under dynamic human-induced conditions. The concept is developed by introducing a Bayesian, dynamic framework (copula) to model time-varying multi-dimensional attributes, and the dependence structure between them, over time. In the framework, the parameters of the model are allowed to change over time to integrate the effect of dynamic climate change-induced conditions.

Since drought is a complex multi-dimensional hydro-climatic phenomenon, and affects a large number of people^21^, this natural phenomenon is selected as an illustrative climate example. Drought attributes consist of dependent variables including, drought severity, drought duration, and the inter-arrival time expressing the recurrence interval of droughts. The impact of climate change on the behaviour of multi-dimensional droughts in twenty large populated cities in Iran is examined using some pre-processing tests (Methods Section). The capital of the country, Tehran, with a population of more than 14 million, is selected as an illustrative domain area. All drought attributes in this city are significantly altered under the impact of climate change. Long-term decision making for water systems in a changing world needs long-term information of the future climate change impacts. To obtain this information, historical observed records are synthetized with future climate model projections from multiple models^3^,^22^ (Methods Section). In this procedure, using multi-climate model ensembles helps water resources planners identify sources of uncertainty and measure the degree of climate change dynamic influence on multi-dimensional extreme events in the future^23^,^24^. Fifteen climate models of the IPCC fifth assessment report (AR5), under the worst radiative forcing scenario (RCP8.5), are used to project the impact of climate change on the drought multi-attributes in the twenty-first century. The results of pre-processing trend tests indicate that climate change is expected to alter the behaviour of extreme multi-dimensional droughts (Table S1 in Supplementary Material). The presence of sustained changes (non-stationarity) in extreme multi-dimensional droughts over time violates the fundamental assumption of currently-used multi-dimensional stationary risk frameworks, and requires the implementation of the adaptive multi-dimensional time-varying risk framework.

To demonstrate the importance of defining a new concept of risk under changing change conditions, a comparison is carried out on outputs of two risk frameworks (non-stationary and stationary). In the static stationary risk theory, it is assumed that the probabilistic properties of extreme multi-dimensional events are independent of time. In the time-varying risk framework, the parameters and properties of the underlying joint attributes are non-stationary and change over time. The fully time-varying multivariate risk framework is updated by adding information from annual observations and projections over time. Depending on the context, the updating temporal resolution can be defined in the finer or coarser scales. Figure 1 illustrates the uncertainty that arises from a static stationary and an adaptive time-varying risk framework for droughts under projections of a climate model (synthetized INMCM4) over a design’s life period (2015–2100) as an example. As the motion of the time-varying risk cannot be shown over time in a 2D plot, three time frames are selected from the frameworks to compare the multi-dimensional risks. At the beginning of the design period (2015), the stationary-based framework substantially overestimates the risk of multi-dimensional historical drought occurrences in comparison with the non-stationary framework for the same time. For example, all drought observations occur in return period of less than 50yr in the time-varying framework, while the same droughts occur in return period of less than 20yr under the static framework. As time goes by over the design’s life period, the nature and the risk of multi-dimensional droughts are changed under the non-stationary framework. For instance, multi-dimensional droughts, which occur with return period of 20–50yr seen in the previous time slice, no longer exist by the end of the half century (2065). The same droughts will become much more frequent, and will occur with less recurrence interval time. By the end of the 21^st^ century, the same characterized droughts will severely intensify, and become even much more frequent in the non-stationary framework. The assumed risk is static, and not changed substantially over the entire century under the currently-used static framework. To better compare the concept of risk in the two frameworks, an example is given in Fig. 1. An assumed severe drought event (illustrated as a black star) represents joint multi-dimensional attributes of the severity 14.0 and the duration of 19.0 months. The results indicate that under the currently-used static risk framework, droughts having the same joint multi-dimensions constantly occur in return period of 50yr without any changes over the entire design period. According to the time-varying framework in the dynamic condition, the same characterized drought event having a joint return period of about 100yr, occurs less frequent than the drought in the static framework at the beginning of the century (based on historical observations). Such overestimation of the risk (joint return period) in the static framework raises infrastructure design costs and complicates the management of water systems, which is not necessarily based on an actual adaptive time-varying risk framework. The nature of the joint characterized droughts, changes over time under the dynamic condition and becomes much worse in comparison with the static framework. As a result, the same severe multi-dimensional drought becomes much more frequent and occurs in return period of almost 10yr at the end of the century. The results indicate that, after the 2030s, the stationary framework will be underestimating the risk of projected extreme multi-dimensional droughts. That means if the nature-changing assumption of extreme multi-dimensional droughts is overlooked, and such a static-based risk framework is used for water system designs, the systems may not withstand drought conditions as the actual risk is higher than the system design allows. Similar behaviour is observed for the other synthetized climate models (see Figure S1 in Supplementary Material). The results demonstrate that the essence of the complex multi-dimensional droughts is dynamic under the altered environment that arises from climate change. The risk of their occurrence, subsequently, will also become dynamic over time. This fact is demonstrated in Movie S1 (in Supplementary Material), which shows the increase of the time-varying risk for the extreme multi-dimensional droughts.

The proposed multivariate time-varying risk framework offers a number of unique advantages. The procedure performs joint Bayesian inference for conditional copula model describing time-varying dependence between the drought marginals over time. The Bayesian framework makes inference to estimate time-varying parameters by generating samples from a joint distribution through iterative sampling from the full conditional distributions (Table S2). Using Bayesian inference, different formats of trend in the multi-attributes are also captured through time. Furthermore, the credibility of the Bayesian predictive intervals can be developed providing information about the precision of the estimates (Figure S2 in Supplementary Material). The other unique feature of the multivariate time-varying risk framework is the computation of the uncertainty in terms of Joint Return Period (JRP) using time-varying multi-attributes (marginal, copula, and inter-arrival time) in the changing environment against static condition. Figure 2 illustrates the uncertainty in the estimation of joint return period over time in the two stationary and non-stationary based multivariate risk frameworks. The results are shown for a historical event that occurred in 1955 (with the severity *S* ≥ *s* = 11.5 and the duration of *D* ≥ *d* = 11 months). As illustrated in Fig. 2, under the fully nonstationary risk framework, the mean joint return period of such a multi-dimensional event changes over time. The results indicate that the mean joint return period will become less in the future, and such an extreme drought will occur frequently by the end of the century. In the stationary-based risk framework, the mean joint return period of such an extreme event is assumed static without any changes over the entire lifetime. This inability of the currently used multivariate stationary risk framework in capturing actual changing joint return period may lead to high uncertainty and failure of risk plans in water resource systems.

Under the changing climate, the safety and the security of water infrastructure is influenced by the risk of extreme hydro-climatic phenomena. Potential global warming-induced-changes on the multi-dimensional hydro-climatic processes call into question the reliability and the accuracy of the currently-used static stationary-based design concepts. Increasing scientific evidences of the impact of climate change on hydro-climate processes around the world^10^,^25^,^26^,^27^,^28^ implies the fact that the nature of these processes is being changed, and we are facing extreme time-varying multi-dimensional events. It is of crucial importance to therefore update the currently used static based design concepts and develop new generation of the adaptive multi-dimensional time-varying-based methodologies for future risk-based designs. The current study outlines a novel framework of a multi-dimensional time-varying risk concept to be incorporated in updating infrastructure design strategies under dynamic environments arising from climate change. The time-varying multivariate risk concept can be effectively used for infrastructure designs and water resources planning. Using the dynamic risk strategy, one is able to estimate in what extent the probability of exceedance associated with extreme multi-dimensional climate event occurrences may change under the intensified non-stationary conditions. Knowing that, engineering designers can reduce uncertainties in the estimation of risk, which results in enhancing reliability of water resources infrastructure designs and reducing the potential costs. In terms of water resources planning and management, using the multivariate nonstationary risk framework, one is also able to project the time-varying frequency and magnitude of multi-dimensional extreme events under the impact of climate change. In this case, ensemble IPCC model runs can be used as an application for future projection of multi-dimensional extreme events in near and long-term periods. Perceiving that, water managers can better operate water supply systems in a wise way to deliver the water that is demanded without any failure under a complex and uncertain changing climate. The time-varying risk can be designed for any fine or coarse temporal resolutions. Depending on the context that the time-varying multivariate risk framework is developed for, it can be updated over time by adding new information (observations) for the same time-scale.

Updating long-term risked-based water resources decision-making strategies requires interdisciplinary cooperation of engineers, policy makers, climate experts, and economists. We believe that the current static-based risk concepts should be replaced with the proposed multi-dimensional time-varying risk one. Further research should be dedicated to apply and develop the proposed framework in other disciplines that are under the influence of changing environments.

## Methods

The multi-dimensional attributes of drought are defined based on the Standardized Precipitation Index (SPI) measure formed by monthly precipitation time series under the run theory^29^. Relatively short historical precipitation records, and the uncertainties associated with future precipitation projections from climate models, are considered major restrictions for designing in a future changing climate. To address these limitations, and to quantify future extreme dry-spells, short-term historical precipitation records from 1951 to 2015 are combined with future long-term precipitation time series. Future projections are derived from the most recent Coupled Model Intercomparison Project Phase 5 (CMIP5) climate multi-model ensemble using statistical downscaling over the design’s life period (2015–2100)^30^. Climate multi-model ensembles allow the measurement of probabilistic uncertainties that arise from climate change, and better communicate certainty of extreme event occurrences. Note that future long-term precipitation time series are projected, and verified in a retrospective statistical downscaling framework after implementing a bias correction process^31^. The combined historical and future projected precipitation time series from 1951 to 2100 from different CMIP5 multi-model ensemble under RCP8.5 emission scenario are used to form the multi-model ensembles of the SPI time series. The joint drought attributes, and the inter-arrival time characteristic, are extracted from each multiple SPI time series. To examine the presence of the dynamic, non-stationarity condition that arises from climate change, univariate and multivariate Mann-Kendall trend analyses^32^ are applied. The tests are employed on the marginals (drought severity and drought duration) and the dependence structure (copula) between the correlated marginals.

Upon detection of trends using a multivariate Mann-Kendall (MK) test, a dynamic copula allows a time variant dependence structure to characterize the relationship of underlying multi-dimensional attributes in a more flexible and dynamic manner. Suppose that *y*_*t*_ = (*y*_1*t*_, *y*_2*t*_) represents a pair of hydro-climatic variables whose dependence structure is defined by a copula function. A time varying joint distribution can be developed at any time *t* using a dynamic copula^33^:


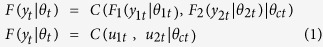


where *F*(.) denotes cumulative distribution function, *C*(.) is the copula function, θ_1t_ and θ_2t_ are parameters of the time varying marginal models, and 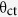 is the time varying copula parameter. u_1t_ and u_2t_ are also marginal probabilities in the dynamic copula in the unit hypercube with uniform marginal distributions U[0, 1].

In the present study, marginal distribution parameters and copula function are specified as functions of time (as a covariate). The parameters are estimated via the Generalized Linear Model (GLM), which captures different forms of linear and nonlinear trends in terms of location parameter (*μ*_*t*_) in the marginal distributions. Different types of time-varying distributions can be selected for the drought severity^29^,^34^. Assume *S*_*t*_ is the severity attribute starting at real time *t*. Then one of the potential time varying model possibilities will be Gamma model:


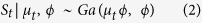


Then different forms of constant, linear, and quadratic models are considered in terms of location parameter (*μ*_*t*_) as follows:


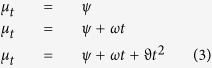


Therefore, a set of the severity model parameters is given by β_S_ = (ψ, ω, ϑ, ϕ).

Since drought duration is a discrete attribute, d ∈ {1, 2, …}, we consider time varying discrete distributions. For example, assume the Negative Binomial distribution is one of the possibilities fitting to 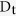, the duration starting at real time t. Then the dynamic model is given by:


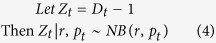


where *p*_*t*_ = *r*/(*r* + *λ*_*t*_), and *λ*_*t*_ is the rate of drought occurrence. The same forms of non-stationarity used for the location parameter in the severity model are also considered for log *λ*_*t*_. The set of duration model parameters is thus given by β_D_ = (η, θ, κ, r). The similar structure can be used for other potential continuous and discrete distributions to be fitted to the multi-attributes.

Let C_t_(u_t_, v_t_) represent the dependence structure between mixed outcomes of severity and duration for a drought that begins at real time *t*. A dynamic Gumbel copula C_t_(u_t_, v_t_)|*θ*_*ct*_ is developed for drought observations, as this exhibits greater dependence in the positive tail than in the negative. It is therefore an appropriate choice for extreme multivariate analyses^14^,^35^. The dynamic Gumbel copula is then given by:


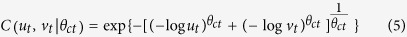


where *θ*_*ct*_ ∈ [1, ∞]. The relation between copula function *θ*_*ct*_ and the standard, Kendall’s tau dependence values can be expressed as τ_t_ = 1 − 1/*θ*_*ct*_, where τ_t_ is always in (0, 1). To capture different forms of Kendall’s tau under changing non-stationary conditions, the following models are assumed:


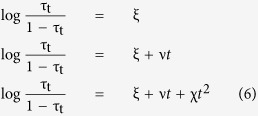


where β_C_ = (ξ, *v*, χ) are the copula model parameters.

As with the evolution of a drought, inter-arrival time between events may vary over time, the behaviour of this variable may be dynamic as well. This attribute is thus modeled through similar potential discrete distributions fitted to the duration dimension. For example a possible dynamic Negative Binomial inter-arrival model will be:


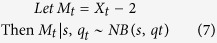


where *q*_*t*_ = *s*/(*s* + *γ*_*t*_). In this case, a set of inter-arrival model parameters is given by 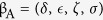. Taking into account the non-stationary inter-arrival times, a time-varying Joint Re*t*urn Period at time *t*, denoted by *JRP*_*t*_(*d*_0_, *s*_0_) is developed as follows:


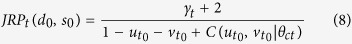


To characterize the uncertainty of the proposed multivariate time-varying risk framework, a Bayesian Markov Chain Monte Carlo (MCMC) approach is integrated to the non-stationary marginal and copula models. Instead of directly inferring the time varying distribution parameters, μ_t_, λ_t_, *θ*_*ct*_ and γ_t_, Bayesian inferences are employed to estimate the GLM parameters β_S_, β_D_, β_A_ and β_C_, respectively, linking the parameter values at time *t* with the time as a covariate. Bayesian inference defines prior distributions for all unknown GLM parameters. The knowledge brought by a prior distribution is combined with the given observations to generate posterior distribution by Bayes theorem. To estimate parameters inferred by Bayes, a Gibbs MCMC sampling method is integrated to generate an approximate Monte Carlo sample of realizations from the posterior distributions. To assess the convergence of the Markov chain, the *Geweke* method is employed^36^. Once samples of the posterior distributions for the parameters of the different trend models (including, constant, linear, and quadratic) are obtained, the Deviance Information Criterion (DIC)^37^ is used to select the best fitted non-stationary model. Note that in the case of stationarity, parameters of the trend models are equal to zero and the parameter μ_t_ remains constant, and consequently observations are independent and identically distributed.

## Additional Information

**How to cite this article**: Sarhadi, A. *et al*. A New Time-varying Concept of Risk in a Changing Climate. *Sci. Rep.* **6**, 35755; doi: 10.1038/srep35755 (2016).

## Supplementary Material

Supplementary Document

Supplementary Movie S1

## Figures and Tables

**Figure 1:**
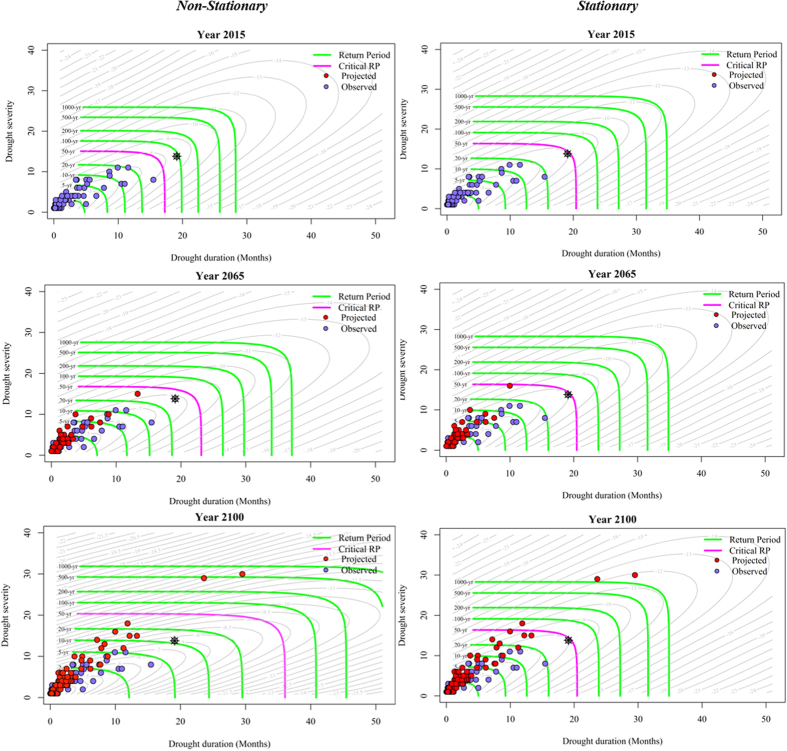
Non-stationary (time-varying) vs. stationary (static) joint return periods for three time slices of the INM-CM4 model. The essence of droughts is dynamic, and drought characteristics are changing over time in the non-stationary condition. In the stationary condition, the risk of droughts is constant over the time period. The risk changes of the particular assumed drought (symbolized as a black star) are also illustrated in the both frameworks. Gray contour lines illustrate the log density of the Gumbel copula.

**Figure 2:**
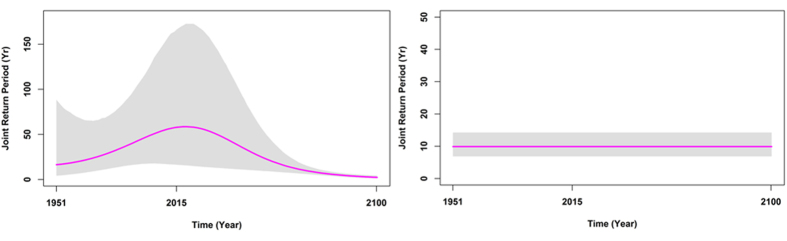
The uncertainty of mean joint return period over time with duration and severity equal to or greater than the 1955 Tehran extreme drought in nonstationary and stationary multivariate risk framework. The shaded area illustrate Bayesian Credible Intervals (2.5% and 97.5%) for the return periods.
