# Supplementary material for: A New Time-varying Concept of Risk in a Changing Climate
Source: Sci Rep. 2016 Oct 20;6:35755. doi: 10.1038/srep35755 (PMC5071900; doi:10.1038/srep35755)
Supplement: Supplementary Document [file srep35755-s1.pdf]

Supplementary Materials

A New Time-varying Concept of Risk in a Changing Climate

Ali Sarhadi*[1], María Concepción Ausín[2], Michael P. Wiper[2]

[1]Department of Civil and Environmental Engineering, University of Waterloo, Waterloo, Ontario, Canada N2L-3G1
[2]Departamento de Estadística, Universidad Carlos III de Madrid, Getafe, Spain

* Corresponding Author: Ali Sarhadi (asarhadi@uwaterloo.ca)

Table S1 | Results of the best selected distributions, and univariate and multivariate Mann-Kendall trend statistics in terms of each drought characteristics for different GCM models under scenario RCP8.5

Attributes	Models	Selected Dist.	Univariate MK	P-value	Multivariate MK	P-value
Severity	**GFDL-ESM2M**	Gamma Distribution	**1.84**	**0.050***		
	GISS-E2-R		-0.712	0.476		
	**INM-CM4**		**1.86**	**0.041***		
	IPSL-CM5A-MR		-0.661	0.508		
	MIROC-ESM		-0.160	0.872		
	MRI-CGCM3		0.143	0.886		
	NorESM1-M		1.348	0.177		
Duration	**GFDL-ESM2M**	Negative Binomial Distribution	**2.75**	**0.005***		
	GISS-E2-R		0.996	0.318		
	**INM-CM4**		**2.47**	**0.013***		
	IPSL-CM5A-MR		1.34	0.179		
	MIROC-ESM		1.16	0.244		
	**MRI-CGCM3**		**1.83**	**0.050***		
	**NorESM1-M**		**2.390**	**0.016***		
Copula	**GFDL-ESM2M**	Gumbel Copula			**2.35**	**0.011***
	GISS-E2-R				0.133	0.893
	**INM-CM4**				**2.21**	**0.024***
	IPSL-CM5A-MR				0.333	0.739
	MIROC-ESM				0.506	0.612
	MRI-CGCM3				1.002	0.316
	**NorESM1-M**				**1.912**	**0.050***
Inter-Arrival Time	**GFDL-ESM2M**	Negative Binomial Distribution	**-2.59**	**0.009****		
	**GISS-E2-R**		**-2.13**	**0.032***		
	**INM-CM4**		**2.21**	**0.022***		
	**IPSL-CM5A-MR**		**-1.91**	**0.050***		
	**MIROC-ESM**		**-2.07**	**0.037***		
	MRI-CGCM3		-0.439	0.660		
	NorESM1-M		-0.529	0.596		

* 5% Significance level
** 1% Significance level

Table S2 | Uncertainty assessment of time varying distribution parameters of the drought attributes estimated through the posterior distribution of the MCMC samples in the non-stationary and stationary conditions. Considering a trend imposes a certain type of non-stationarity, outputs of posterior distributions should be checked to select the best statistical model capturing the non-stationarity form. Three different modes of the posterior distributions are employed in the parameter estimations to select the best fitted model. 1. Model 0 (M0): this model is used when non-stationary model does not fit to the model, indicating the mean parameter of distribution (μ_t) or dependence (θ_{ct}) is time invariant. Model 1 (M1): This model is accounted for a non-stationary condition by assuming the model parameter is a linear function of time. Model 2 (M2): In this model a quadratic function is used to model the non-stationarity in the distribution parameters and dependence structure. The least Deviance Information Criterion (DIC) is used to select the best fitted non-stationary model.

**Attributes**	**Models**	**Statistical Model**	**DIC**	**Parameters (Non-Stationary)***	**Parameters (Stationary)**
Severity	**GFDL-ESM2M**	**M2**	772.9	2.82	3.07
	GISS-E2-R	**M2**	759.6	3.24	2.95
	**INM-CM4**	**M2**	626.53	2.94	2.74
	IPSL-CM5A-MR	**M2**	674.6	2.97	3.6
	MIROC-ESM	**M2**	704.2	3.88	4.26
	MRI-CGCM3	M0	682.8	4.40	4.40
	NorESM1-M	**M2**	733.0	3.59	3.30
Duration	**GFDL-ESM2M**	**M2**	890.26	2.89	2.76
	GISS-E2-R	**M2**	876.2	2.64	2.82
	**INM-CM4**	**M2**	737.39	3.14	3.15
	IPSL-CM5A-MR	**M2**	813.4	3.04	3.01
	MIROC-ESM	**M1**	794.3	3.36	3.64
	**MRI-CGCM3**	**M1**	763.3	3.76	4.35
	**NorESM1-M**	**M2**	837.7	2.68	3.25
Copula	**GFDL-ESM2M**	**M2**	-249.63	2.93	2.89
	GISS-E2-R	**M0**	-249.09	2.86	2.75
	**INM-CM4**	**M2**	-200.40	2.98	2.77
	IPSL-CM5A-MR	**M0**	-186.27	2.55	2.57
	MIROC-ESM	**M1**	-174.3	2.99	2.77
	MRI-CGCM3	**M2**	-175.05	2.69	2.91
	**NorESM1-M**	**M0**	-203.29	2.66	2.84
Inter-Arrival Time	**GFDL-ESM2M**	**M2**	915.48	3.52	3.61
	**GISS-E2-R**	M0	919.6	3.20	3.20
	**INM-CM4**	**M2**	811.48	4.11	3.96
	**IPSL-CM5A-MR**	**M2**	861.2	3.58	3.40
	**MIROC-ESM**	**M2**	847.8	4.25	3.62
	MRI-CGCM3	**M2**	815.5	4.16	5.31
	NorESM1-M	M0	890.65	3.41	3.41

* The results are the mean of the estimated parameters based on posterior distributions from the Bayesian model

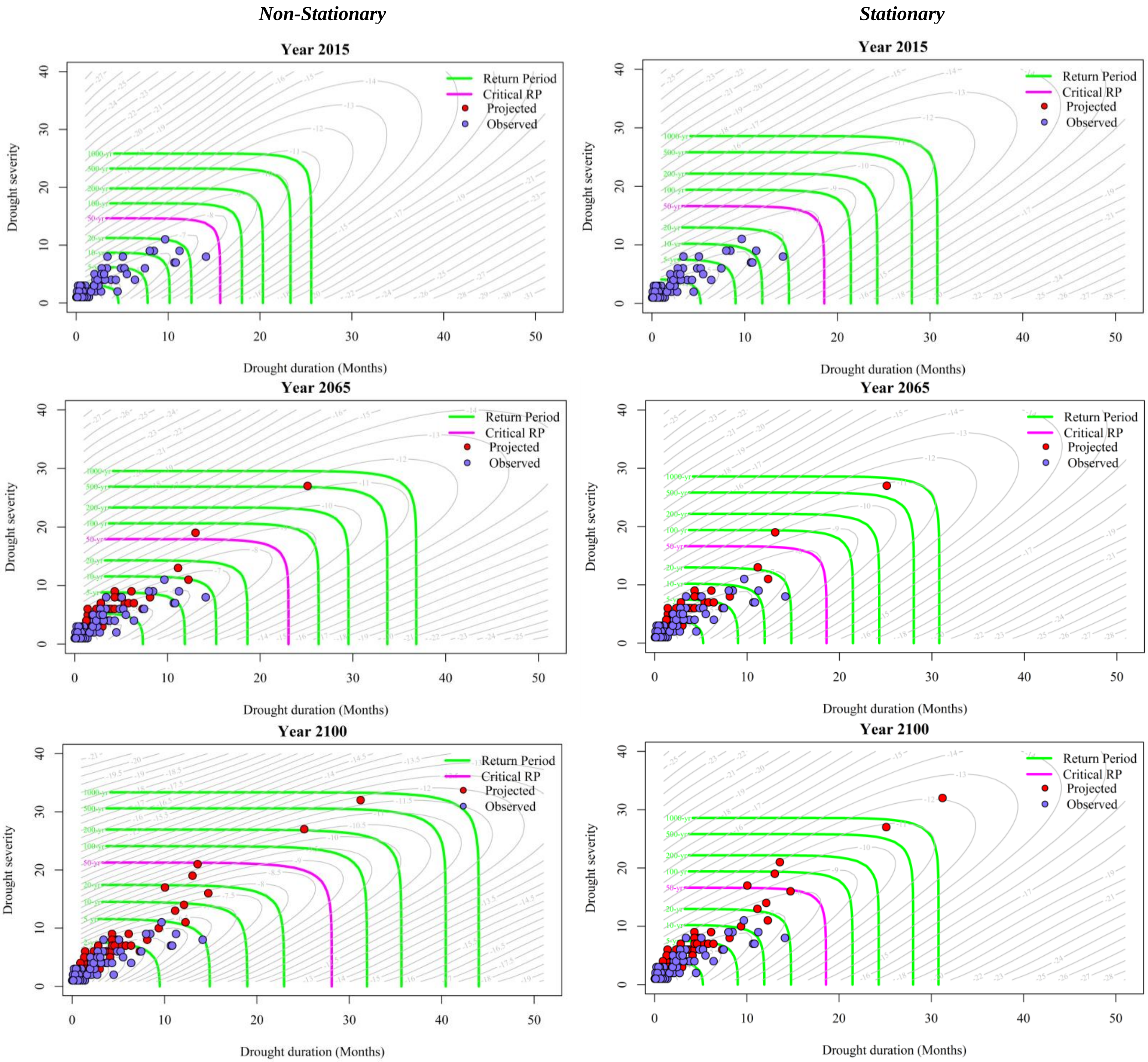

Figure S1 | Non-stationary vs. stationary joint return periods for the three time slices of the GFDL-ESM2M model. Similar to the behaviour of the INM-CM4 model, the essence of droughts is dynamic and drought characteristics are changing over time in the non-stationary condition for this model as well. In the stationary condition, however, the risk of droughts is constant over the time period.

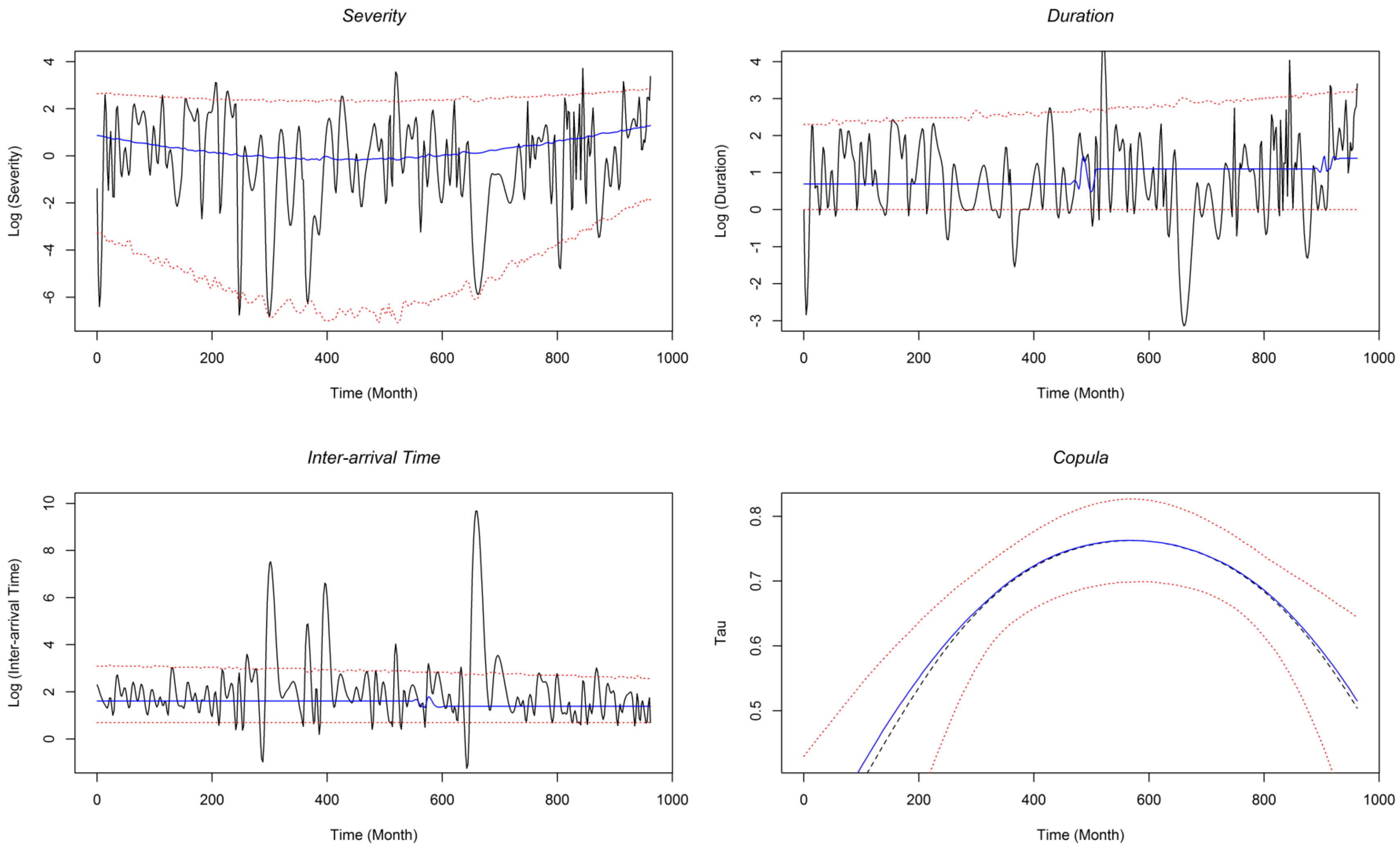

Figure S2 | Predictive mean (blue solid line), true mean of Kendall's τ_t (black dashed line), 95% Bayesian confidence intervals (red dotted lines), and drought attribute time series (black solid lines) for the fully dynamic illustrative model (INMCM4) under forcing scenario RCP8.5

Supplementary Movie S1 | Changes of the nature and risk of multi-dimensional extreme droughts over historical and design's life periods. The blue dots represent observed droughts during historical period (1951-2015), and the red dots represent future projected extreme droughts over the design's life period (2016-2100) based on the INM-CM4 model (as an illustrative GCM example) under scenario RCP8.5. It should be noted that the other multiple ensemble climate models exhibit the same results.
